# Effective impact of nano-plastic-waste incorporated with nanotitina on the physical, mechanical and microstructural properties of white cement pastes composites for progressing towards sustainability

**DOI:** 10.1038/s41598-024-62661-4

**Published:** 2024-05-31

**Authors:** M. A. Abdelzaher, Ahmed A. Farghali, Asmaa S. Hamouda

**Affiliations:** 1 Environmental Science and Industrial Development Department, Faculty of Postgraduate Studies for Advanced Sciences, Beni-Suef University, Beni-Suef, 62511 Egypt; 2 Materials Science and Nanotechnology Department, Faculty of Postgraduate Studies for Advanced Sciences, Beni-Suef University, Beni-Suef, 62511 Egypt

**Keywords:** Industrial coexistence, Nano-plastic-waste (NPW), Nano-Titania (NT), Whiteness reflection (Ry), Physical, mechanical and microstructural properties, Structural materials, Environmental impact, Sustainability

## Abstract

Plastic waste (PW) has received a lot of attention as a possible additional material for industrial and environmental applications, particularly cement and/or concrete production for a more environmentally and economically sound use of raw materials and energy sources. PW has been investigated as an inert and/or active hydraulic filler for cement and/or concrete by numerous scientists. Plastic garbage is cheap, abundant, and takes long period of time to degrade in the eco-system (soil and water). The main goal of the ongoing research is to offer safety and efficacy by partially substituting nano-plastic waste (NPW), incorporated with nano-titania (NT), for the composition of white cement (WC). Blends are built up by substitution of WC with different ratios of NPW incorporated with fixed ratios of nano-titania (1.0 wt.%). Workability, physical, mechanical and microstructural properties have gone through laboratory and instrumental analysis. The results showed improvement in the compressive strength, density and microstructure due to the effective impact of fillers. Consequently, a decrease in total porosity, whiteness reflection (Ry) and early-rapid expansion. Eventually, the outcomes may reduce the pandemic strength, especially in the external environment, and other epidemics.

## Introduction

The manufacture of white cement, start by assembling the raw materials. In this step, the raw materials used in the manufacture of white cement are collected. It is worth noting the necessity of adhering to the specified proportions. Differences in these proportions can cause a difference in the properties of the cement^1^. The process of manufacturing white cement goes through almost the same stages as those used in the manufacture of gray Portland cement, but it is more controlled, and requires the use of carefully selected raw materials, in addition to special manufacturing procedures, to avoid contamination of the product and maintain its whiteness, and deeply explained elsewhere^2^.

PW is abundant in the ecosystem but is rarely recycled or used as a replacement for raw materials in industry. Destruction of both resources, including raw materials and flammable materials, is slowed down by modern waste management procedures. Solid waste has serious harmful effects on both the ecosystem and human health^3–6^. Moreover, a significant amount of plastic trash accumulates as manufacturing levels rise, having an adverse impact on the environment. To reduce its detrimental effects, numerous locally as well as globally regulations have emphasized the necessity to look into waste recycling and landfilling. According to a survey of the literature, recyclable plastic has been the subject of several studies to find environmentally friendly and useful materials^7–9^. A comprehensive investigate how accomplish this on a huge range by preserving the raw materials. Comply with the requirements of cement and concrete specs, due to the enormous increase in popularity of using solid wastes, inexpensive, and hazardous materials in building materials production^10^. Plastic is now widely used in PPE and food packaging, especially over the past five years. Because of this, it is difficult to store or recycle in the traditional ways (such as landfilling and/or recycling) as plastic factory production rises^11–13^. The amount of plastic waste produced in 2025 could total 21 BT^14,15^. Egyptian Mobilization and Statistics center, reported that, PW generation in Egypt's governorates was estimated to be 3716.25 tones between 2020 and 2022^16–20^. Environmental deterioration can be prevented by using this industrial waste that cannot be used in other industries and reaping the financial benefits. Advanced industries like fibers, cultivation, fertilizer, glazing, chemicals, and the building material sector have a remarkable expanding potential for inorganic and affordable materials, such as supplemental cementitious materials (SCMs). PW may also be a useful raw material because of its low cost and availability.

Cement and concrete can benefit from the fantastic approach of nanotechnology and its many uses. Due to their distinctive features, nanoparticles have a variety of functions, including improving the, Physico-mechanical properties and microstructures of cement during addition. Multiple studies on the addition of many nanomaterials to cement and/or concrete have been studied elsewhere^21–26^. Complex physical–mechanical and chemical cement hydration kinetics mechanisms exist^27–30^. Complex mechanisms that thoroughly explain the creation of the C-S-H gel once the hydration process has begun are provided by “*topo-chemical conventional theory*” and the solution's reactions. The methodology of hydration kinetics has 5 main stages, starting with an initial setting period and the growth of a thin protective layer on Alite (C3S) particles. Then, the reaction rate increases till reaching its optimal after less than 21 h, after cement grains mixed with water leads to the growth of Tobermorite gel (C-S-H), and cement matrix become packed more tightly. Finally, the reaction rate gently decreases until it reaches half of its optimal in the remaining^31–33^. Nano-TiO_2_, nano-SiO_2_, or nano-Fe_2_O_3_ have limited uses in nanotechnology. These substances have self-cleaning feature that cause the photocatalytic destruction of airborne contaminants^34,35^. Additionally, nanomaterials behave as active function group during cement hydration, and promote the formation of phases^26,36,37^.

Plastic recycling has great importance and an important role in reducing the exhaustion of sources and achieving sustainable development by securing raw materials from the exploitation of waste instead of raw materials. It also has an important role from an environmental standpoint by protecting the air and water from pollutants by collecting them and reusing them instead of burning, which leads to pollute the air or landfill^38^. Eventually, the use of NPW with NT as an additive for the manufacture of white cement has not been researched yet. Since white Portland cement performs poorly in terms of MCS, water absorption, drying shrinkage, and density, the goal of this study is to determine NPW, employed at different replacement amounts incorporated with fixed TiO_2_ (1.0wt.%); are helpful in improving these characteristics. In summary, WC blend with NPW and NT has been demonstrated to reflect better workability and energy saving qualities, which are economical and environmentally beneficial and may result in decreased construction budget and improve a long-term raw materials sustainability.

## Materials and methods

### Raw materials

**Figure 1:**
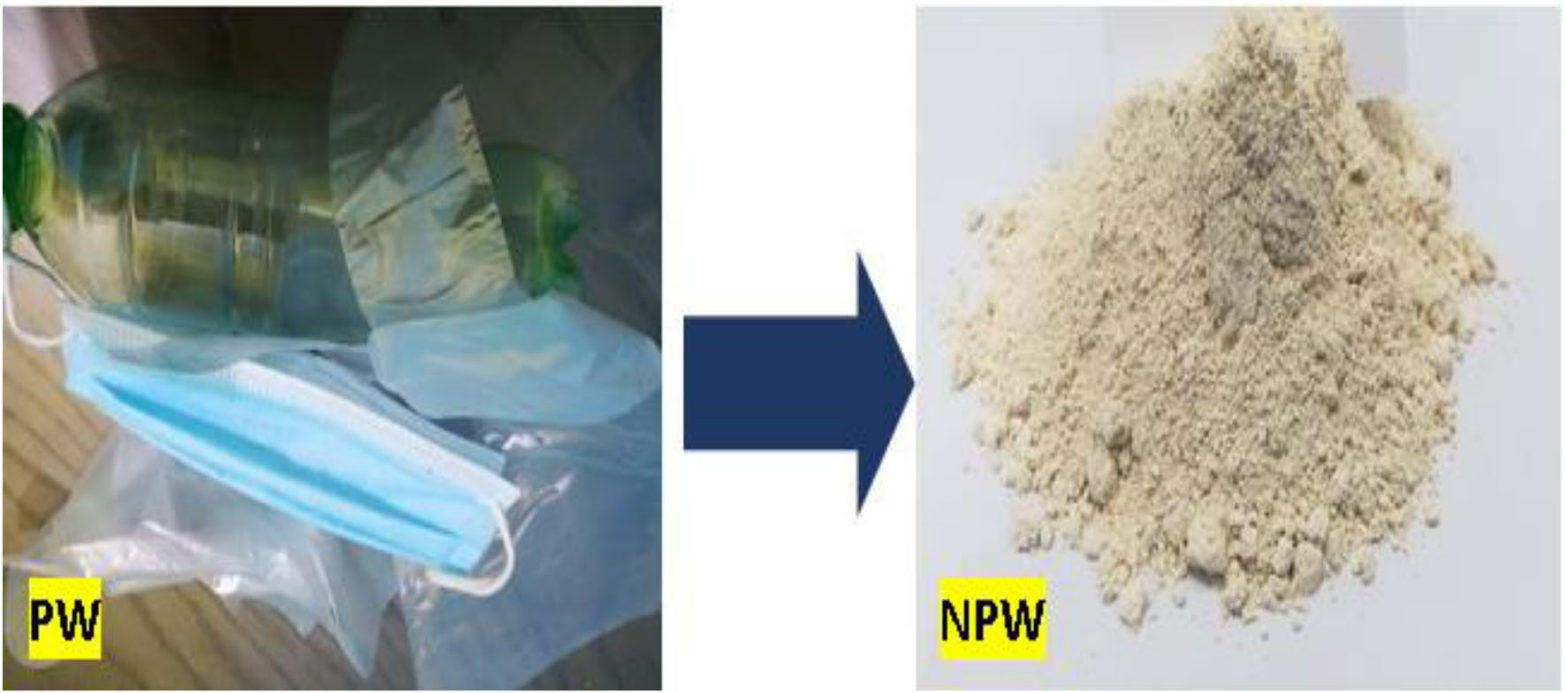
Visual investigation of both PW and NPW^4^.

**Figure 2:**
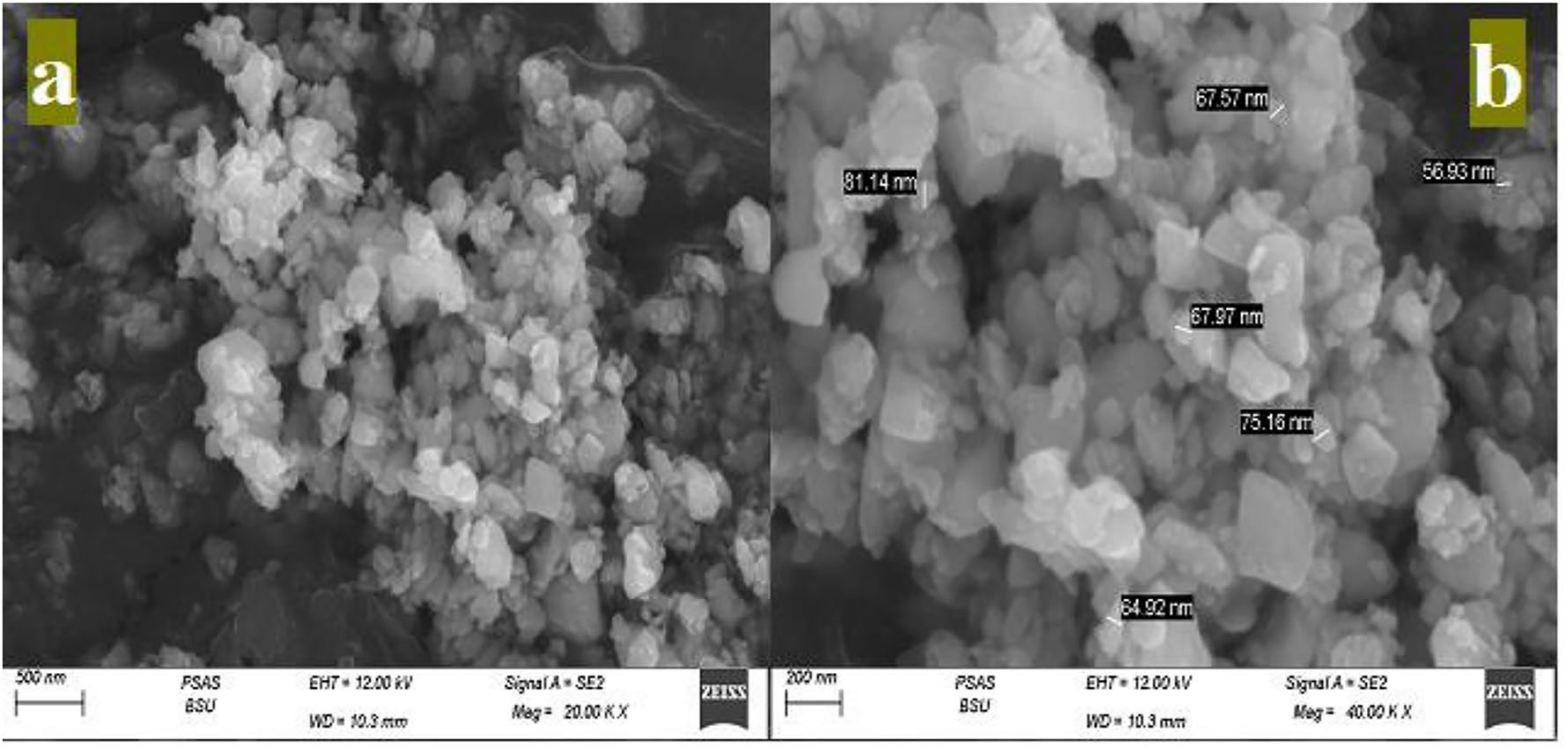
(**a**,**b**) SEM & TEM analysis of NPW^4^.

**Figure 3:**
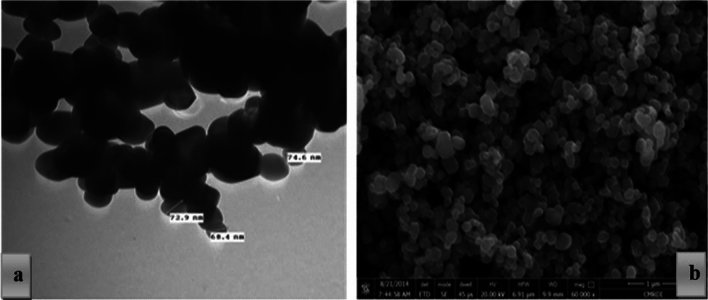
(**a**,**b**) SEM & TEM analysis of NT.

The raw materials used at the current study are, white cement (WC), nano-titania (NT) and nano-plastic-waste (NPW). WC [Class I, 42.5 Pa], purchased from Royal White Cement Company (RCo.), (Minia, Egypt). NT, purchased from Sigma-Aldrich for chemicals company (SACo), (Cairo, Egypt). NPW handled by slicing into small pieces. Plastic is a lightweight, durable, inexpensive and easy-to-modify material. It is made up of polymers, which are large organic molecules composed of repeating carbon units or chains called monomers, such as ethylene, propylene, vinyl chloride and styrene, Fig. 1, shows the small pieces of PW, ground into tiny pieces in a ball mill for 72 h (continuously). The sieved material is examined each 12 h till it reaches micro size. SEM & TEM analysis are reported in Fig. 2a,b, and prove that PW reach nano size scale e.g. NPW size between 56.93 and 81.14 nm., as reported elsewhere^4^. Figure 3 report the SEM & TEM analysis of NT, illustrated the spherical and amorphous shape, while the particle size is on the order between 79 and 99 nm. Table 1, shows the XRF analysis (chemical analysis), for WC and phases compositions e.g. the chemical analysis for WC^21,23^. Table 2 illustrates the features & purity of TiO_2_ nanoparticle.

**Table 1:** The XRF analysis with phases compositions of WC.

Elements	SiO_2_	Al_2_O_3_	CaO	Fe_2_O_3_	MgO	SO_3_	Na_2_O	K_2_O	LOI	Cl-	Sum	C3S	C2S	C4AF	C3A
White cement	23.76	3.21	67.05	0.24	0.37	3.03	0.05	0.05	2.17	0.07	99.44	62.81	20.28	0.729	9.22

**Table 2:** TiO_2_ nanoparticle features.

TiO_2_ nanoparticle	Average diameter/nm	Purity%
	99–79	99

### Preparation and testing methods

Generally, WC has been replaced with different potions e.g. (0.5%, 1.0%, 1.5%, 2.0% and 2.5) by wt. % NPW, incorporated with 1.0% NT, by wt.% (fixed ratio), due to its optimal ratio as discussed elsewhere^24,39^, all proposed mixes are tabulated in Table 3. In addition; W/CP are reported for all MPxT composites. After hand homo-processing, the patches were cast in stainless steel moulds measuring (25 mm × 25 mm × 25 mm), before being placed in cubes to hydrate at a temperature of ambient humidity of 955%. The next day, prisms were de-molded and immersed directly into water for 28 days of hydration^40,41^. In additions; the workability of MPxT blends, Ry, MCS, ST, and porosity, variations had been investigated^42,43^.

**Table 3:** Composition of MPxT blends (As replacement).

Blend composition	WC by weight	NPW by weight	NT by weight	Total by weight	W/CP by weight
M0	100.00	0.00	0.00	100.00	0.35
MP1T	98.50	0.50	1.00	100.00	0.40
MP2T	98.00	1.00	1.00	100.00	0.41
MP3T	97.50	1.50	1.00	100.00	0.48
MP4T	97.00	2.00	1.00	100.00	0.52
MP5T	96.50	2.50	1.00	100.00	0.64

**Figure 4:**
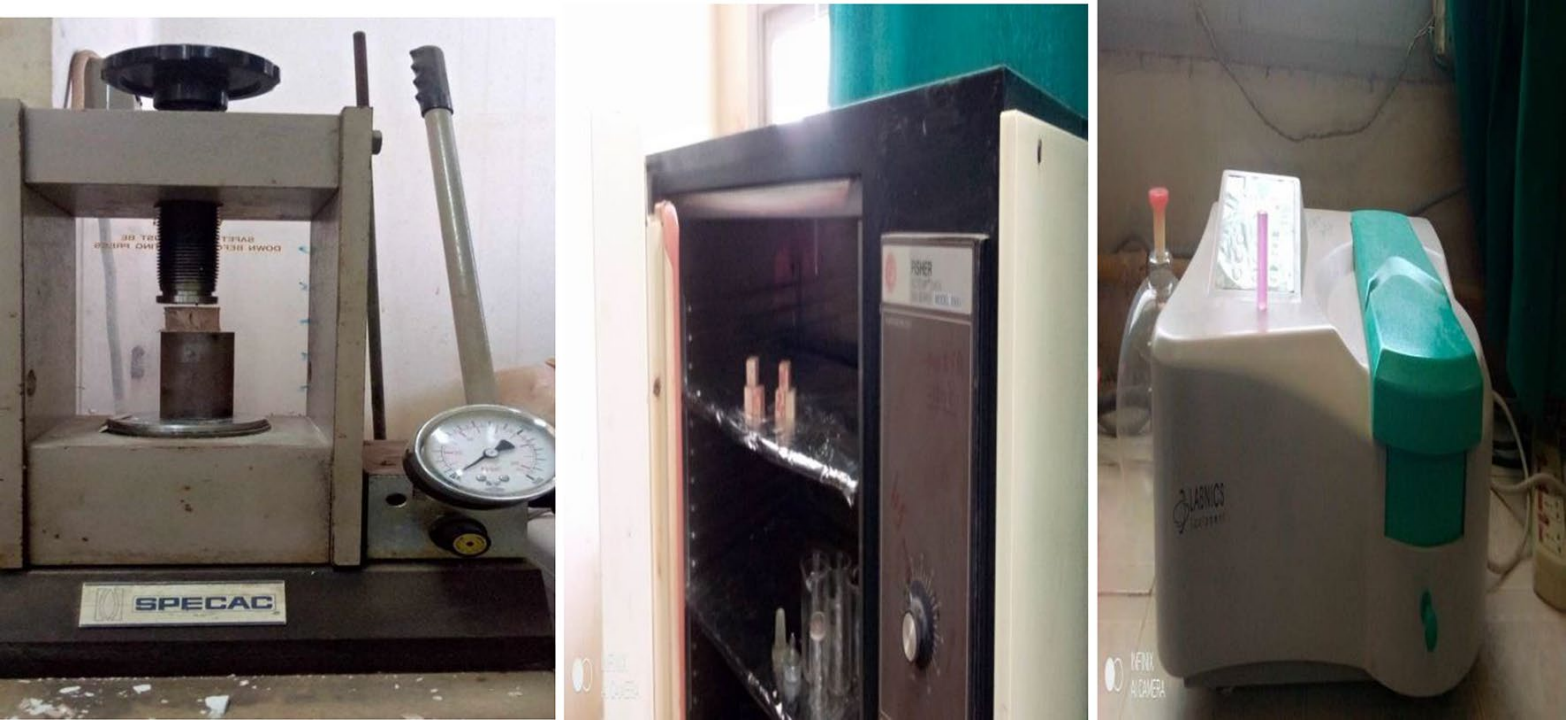
Physical analysis instruments for CSM, ST and expansion (soundness) of MPxT blended cement pastes.

L&W Elerpho apparatus was used to detect the color, brightness, opacity and Ry complies with DIN 5033 specs^44^. Figure 4, shows the physical analysis instruments for CSM, ST and expansion (soundness) of MPxT blended cement pastes measured by Vicate and Le-Chatelier apparatus respectively based on ASTM C191 and ASTM C88 respectively^45^. The MCS was triply performed according to ASTM C109M^46^, by using 5.00 tons’ load (e.g. Shemizitu German machine test) with a high loading rate of 20.00 kg.min^–1^ Solidification of the prisms e.g. porosity percent were conducted from porosimeter (Pore IV 9500), by using mercury intrusion data, this according to the requirements of the standard NF ISO-5017^47,48^. Based on three different weightings, first, the dried sample was weighted (m_1_), after that, the sample is weighted after eliminating the air, using a desiccator, and saturated in a water tank for 72 h: denoted (m_2_). Third, the saturated sample is wiped superficially to remove surface water: denoted (m_3_). Finally, the sample total porosity is given as follow:

1 $$ Porosity = \, \left( {\frac{{m_{3} - m_{1} }}{{m_{3} - m_{2} }}} \right) \times 100. $$

**Figure 5:**
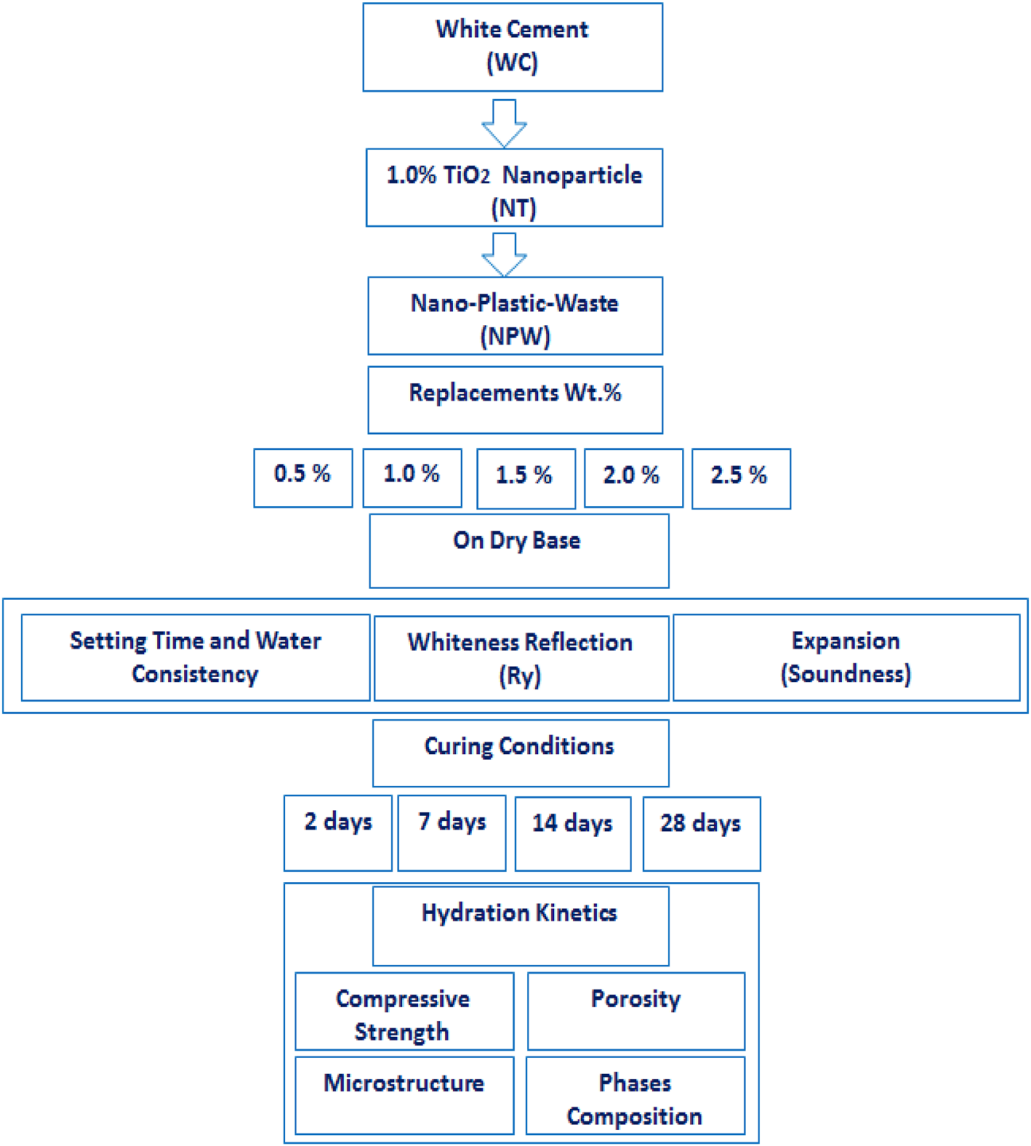
Scientific flowchart for instrumental and experimental program.

According records, the pore sizes (macro-pores large than 3300 nm, micro-pores in 0–15 nm, while meso-pores in range between 15 and 3300 nm) were investigated. The scientific schematic flowchart for experimental program is plotted in Fig. 5.

### Instrumental analysis

Detailed chemical composition analysis for WC performed using XRF (ARL 9900, Panalytical). The morphology of the specimen was conducted with (FEI Company, Netherlands) integrated with EDXA namely *“an energy dissipation X-ray analyzer*”. Another dried sample were pulverized, stored and dried were passed from 90 µm mesh in order to determine the hydration phases using X-ray diffraction (XRD, Philips PW3050/60) diffractometer using a scanning order from 5 to 50 (2Ø), with a scanning speed rate of 1 s.step^–1^ and high-resolution accuracy of 0.05◦step^-1^. The Transmission electron microscopy (TEM) instrument reported that the effective particle size for NPW is 56.93 to 81.14 nm, while NT is 56.93 to 81.14 nm. This indicated that, NPW and NT in nano-size powder, as seen in Figs. 2, 3, which a suitable particle size for mesopores of MPT matrix.

## Results and discussions

### Whiteness reflection (Ry)

**Figure 6:**
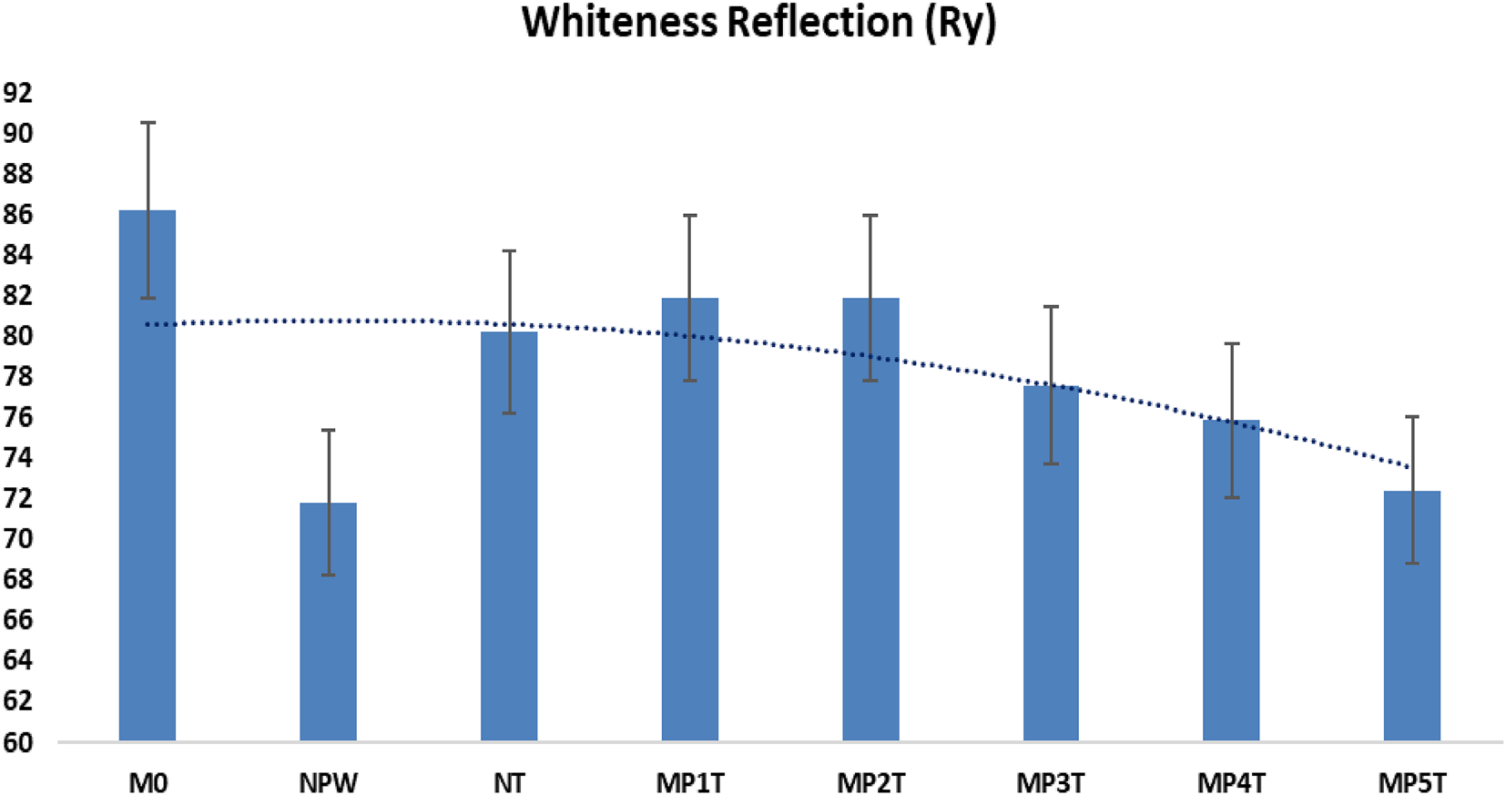
Ry, intensity of MPxT blends.

Replacement will have a negative impact on the WC reflection profile on the 3-axis (Rx = 86.24, Ry = 86.20, Rz = 80.59)^4,44^, since Ry, is one of the key indications for the quality of white cement On the Elerpho apparatus, HL is the key perimeter for the whiteness intensity, whereas Hinter a (Ha) and Hinter b (Hb) are reflections of the colors brown and green, respectively. Under the Elerpho apparatus, both NPW and NT have low Ry intensity. Light yellow color for NPW and low whiteness color for NT diminish MPxT-pastes composites, as indicated in Table 4 (Rx = 71.83, Ry = 71.80, Rz = 67.13 and Rx = 80.24, Ry = 80.20, Rz = 74.98). As shown in Fig. 6, MPxT composite pastes are organised as follows order: M0 ˃ MP1T = MP2T ˃ MPT3 ˃ MP4T ˃ MP5T. Additionally, low Ha and Hb values for NPW (Ha = 1.84, Hb = 3.86), and NT (Ha = 1.95, Hb = 4.08), which result in decreasing the green color and an increase in the yellow color contrast, result in lower Hl values for the blends. Due to the equal 1.0% replacement and neutralization of the Ry color (Ry = 81.89), both MP1T and MP1T exhibit better Hl intensity (Ha = 90.49). Since there is a large amount of yellowish color and a low content of green color, a high replacement of NPW causes a shapely fall in Ha for MP5T paste, erg; (Hl = 85.09). It is advised to partially substitute NPW and NT in equal ratios (1.0 wt%) for clinker/WC.

**Table 4:** Ry, of MPxT blends.

Pastes	Rx	Ry	Rz	Hl	Ha	Hb
M0	86.24	86.20	80.59	92.84	− 2.02	4.23
NPW	71.83	71.80	67.13	84.73	− 1.84	3.86
NT	80.24	80.20	74.98	89.55	− 1.95	4.08
MP1T	81.93	81.89	76.56	90.49	− 1.97	4.12
MP2T	81.93	81.89	76.56	90.49	− 1.97	4.12
MP3T	77.62	77.58	72.53	88.08	− 1.92	4.01
MP4T	75.88	75.85	70.92	87.09	− 1.90	3.97
MP5T	72.43	72.40	67.69	85.09	− 1.85	3.87

### Setting time and water consistency

**Figure 7:**
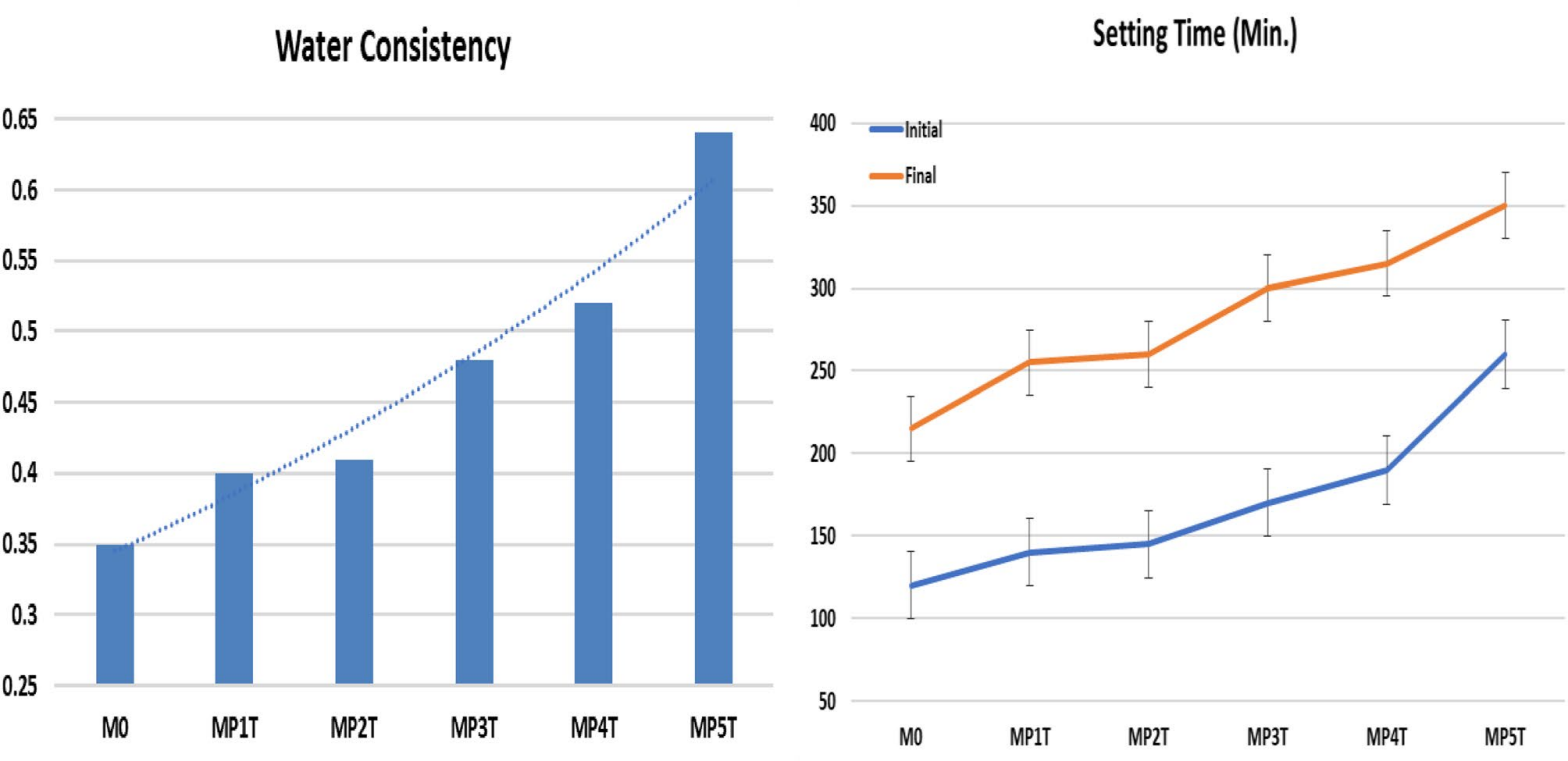
Setting time and water constancy of MPxT blends.

The initial & final STs of the WC-composites are prolonged as the water consistency is increased. While NT is a hydraulic filler, NPW serves as an inert filler. The blends are timed in the following order: Fig. 7 displays M0 (0.35) < MP1T (0.40) = MP2T (0.41) < MP3T (0.48) < MP4T (0.52) < MP5T (0.64). NT is preferred padding because they yield C-S-H and calcium-sulfoaluminate during the pre-hydration process and minimize the acoustic behavior. WC, replacement with the fillers lengthens the cement ST^49^. Due to the similar amounts of inert and hydraulic filler in the MP2T mix, which control the blend setting, MP1T and MP2T exhibit good workability and nearly identical water to cement ratios. Additionally, hydration kinetics prevent early cracking and boost solidification for blends. On contrast, while being on the nanoscale, MPT5 mix exhibits low level of plasticity due to excessive NPW addition. The cement hydration phases behavior is reduced as NPW content is increased. Elevated surface area grains fill the WC matrix's open pores, raising the water requirement. The average water to cement weight ratio for white cement is 0.40 till 0.03, which demonstrates appropriate water consistency and hydration products.

### Expansion (soundness)

**Figure 8:**
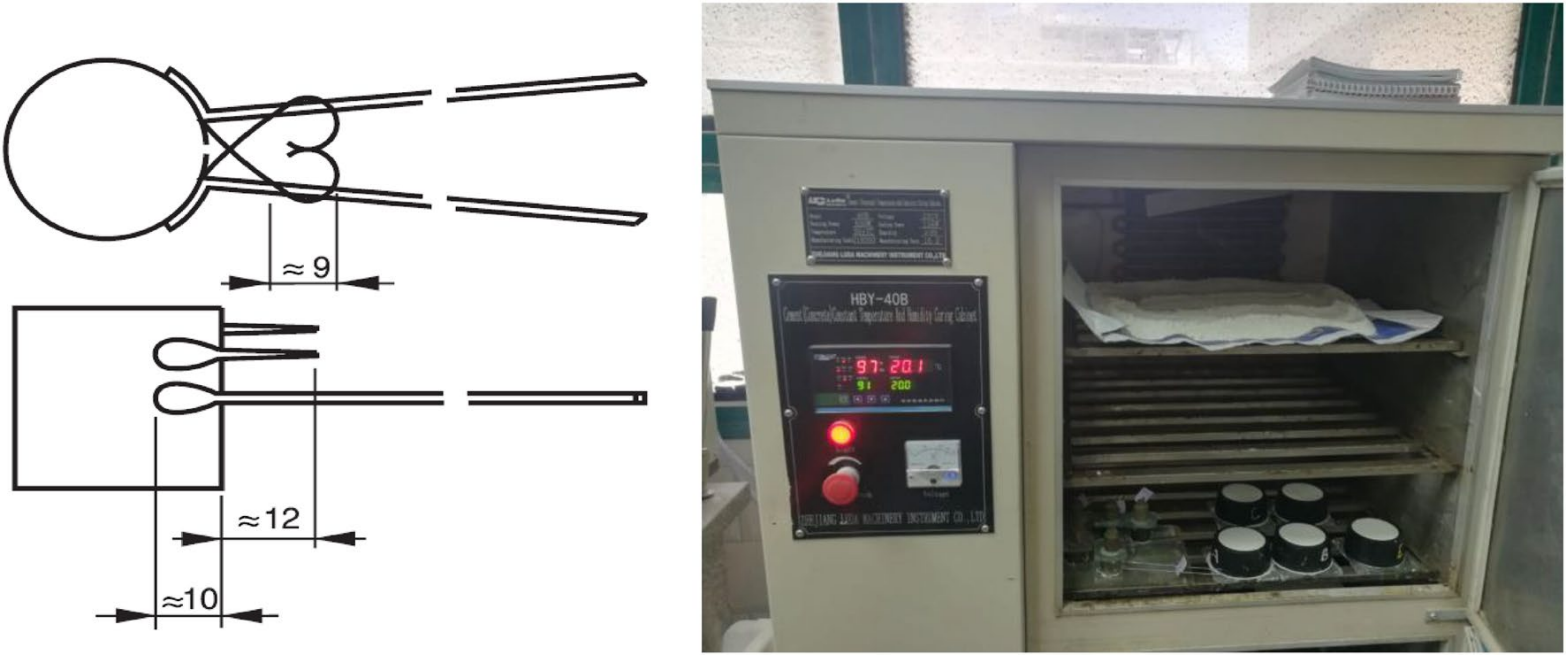
Soundness (Le-Chatelier) test apparatus and cabinet of MPxT blends.

**Figure 9:**
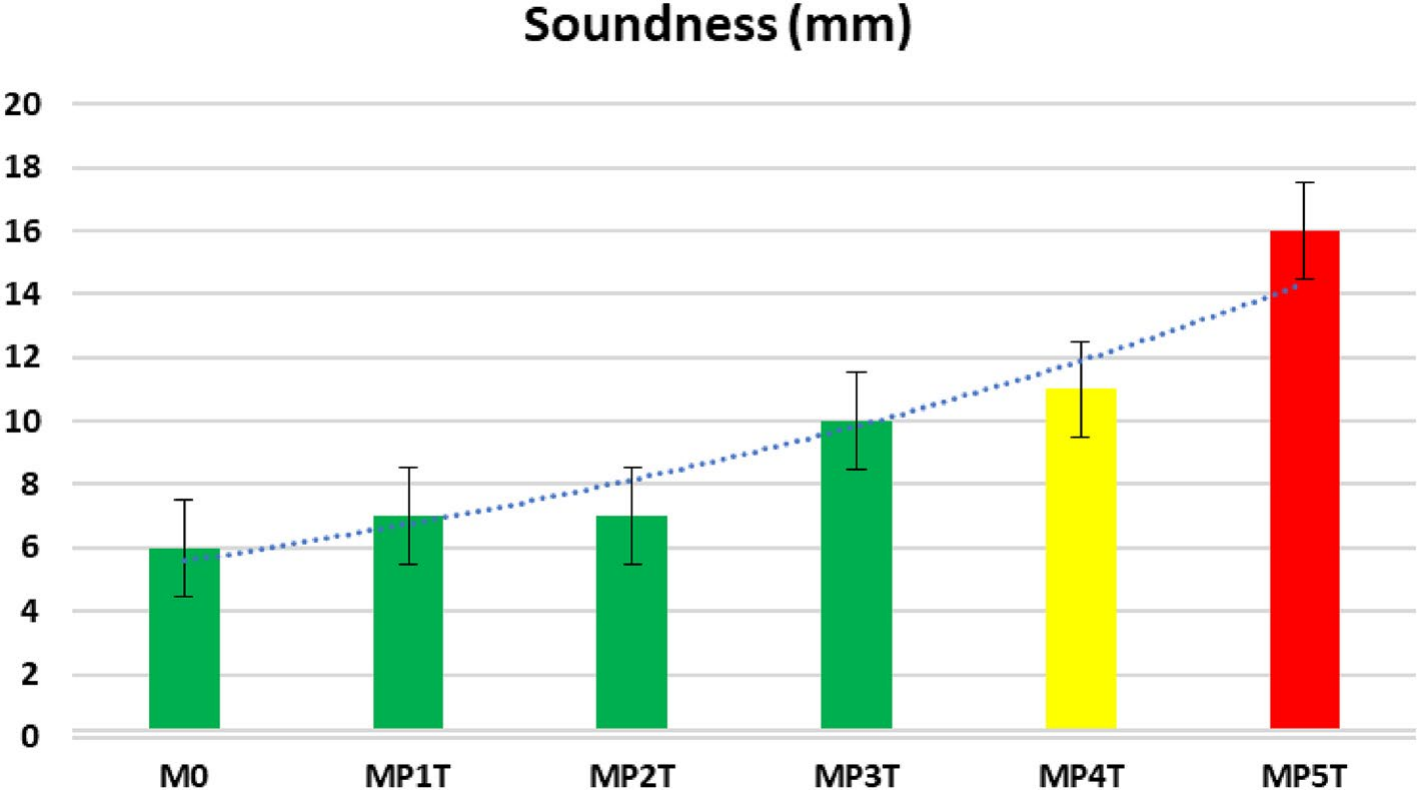
Soundness of MPxT blends.

Soundness; determined in accordance with cement and concrete specifications (≤ 10 mm)^50^. Alkalis and halides are undesired elements in cement composition as they react with free calcium oxide in cement during hydration process yields calcium and magnesium halides leads to cement corrosion and early cracking, the Le-Chatelier test apparatus and cabinet are shown in Fig. 8^51–53^. The blends are in the following order: M0 (6 mm) < MP1T (7 mm) = MP2T (7 mm) < MP3T (10 mm) < MP4T (11 mm) < MP5T (16 mm), as reported in Fig. 9. Dynamic fillers promote the formation of C-S-H and calcium-sulfoaluminate during the hydration process and minimize the acoustic behavior. 2.0 wt.% replacement from NPW leads to increase the expansion, and consequently 2.5 wt.% replacement from NPW promote the expansion to negative direction. Both blends MP4T and MP5T are not comply with the stander, hence not recommended replacement^54^.

### Mechanical compressive strength (MCS)

**Figure 10:**
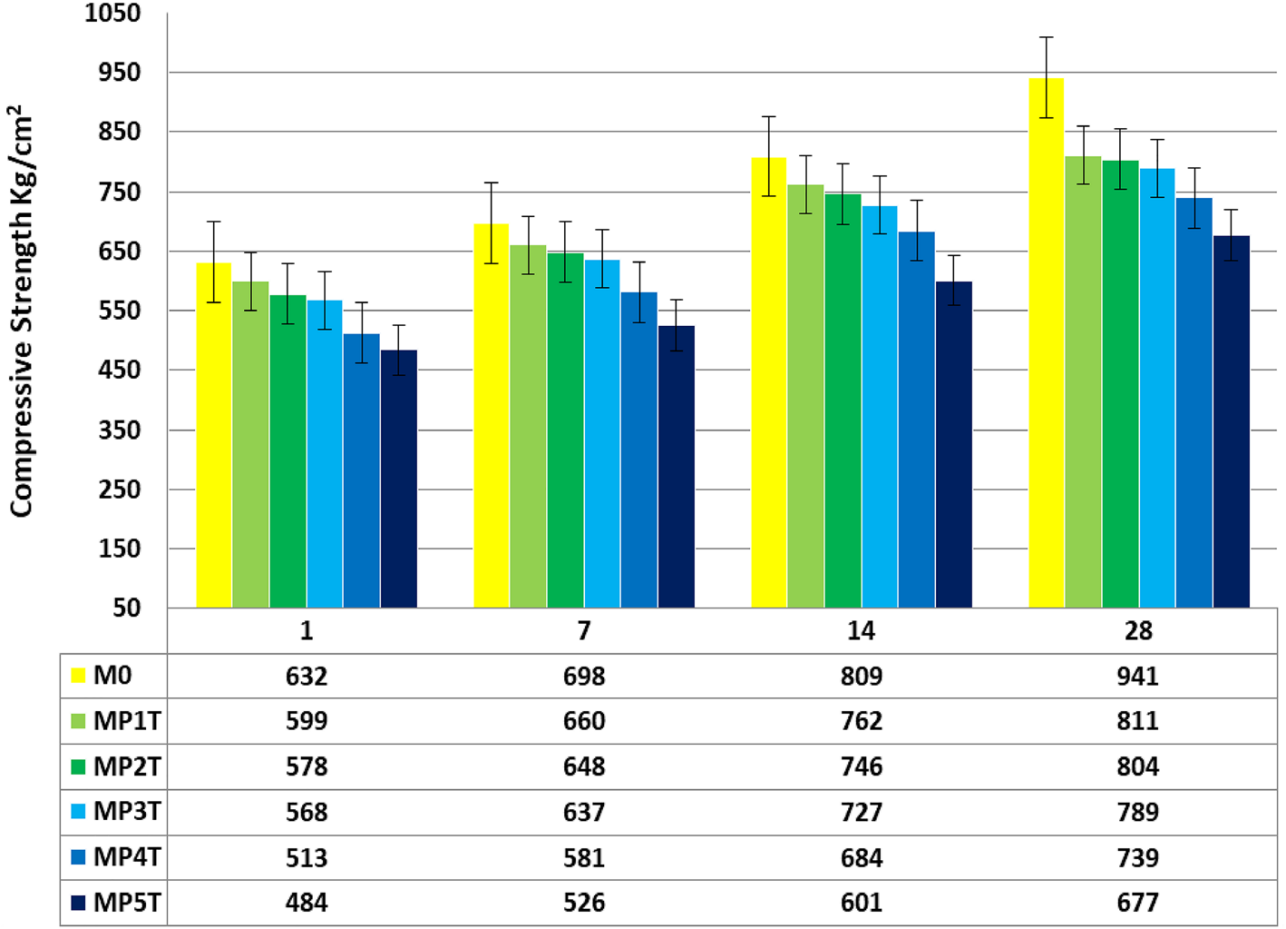
MCS of MPxT blends.

Figure 10 illustrates the impact of NT and NPW addition on the MCS of the composite throughout a late hydration period as a function of hydration ages. According to the data, as more C-A-H and C-S–H (solidification phases) continue to solidify and are completely deposited inside the open pores, the MCS of all blends increases as setting times lengthen. This results in an improvement in the compactness of the specimen microstructure^55^. The MCS of the NT-composite were improved by the addition of NT (1.0%); this attributed to NT, acts as active dynamic filler, improving the microstructure of the blends, whereas NPW has an inert effect during the hydration process. The gels C-S-H, C-A-H, and C-A-S-H that comprise the compacted microstructure of the hardened WC matrices are created by the interaction of hydration between NT and free lime and calcium hydroxide^56^. However, adding NT is recommended to a particular amount (1%) because anything higher than this could damage the composite's microstructure and have an adverse effect on the matrix. The outcomes also showed that the control sample's MCS was higher compared to WC composites incorporated with 1.0% from both NPW and NT. The MP1T sample is reduced while the bulk density is increased by the NT. In comparison, the MCS is measured in the manner shown below: When the ratio of NPW & NT incorporated with WC pastes reduced, which is attributable to a decrease in the hydraulic activities of the cement pastes composites, M0 ˃ MP1T ˃ MP2T ˃ MP3T ˃ MP4T ˃ MP5T. Blends hydrated to 2, 7, 14, and 28 days, respectively, the MCS of the composite has of 98.5% WC and 0.5% NPW + 1.0% NT is higher than other pastes by 1, 5, 11, and 17% respectively. The high Na_eq_ content of WC is caused by the high alkaline component of WC, which includes both Na^+^ and K^+^. These alkalis create Na^+^-CSH and K^+^-CSH, which increase the CS by around 7.0%^57^.

### Total porosity (TP)

**Figure 11:**
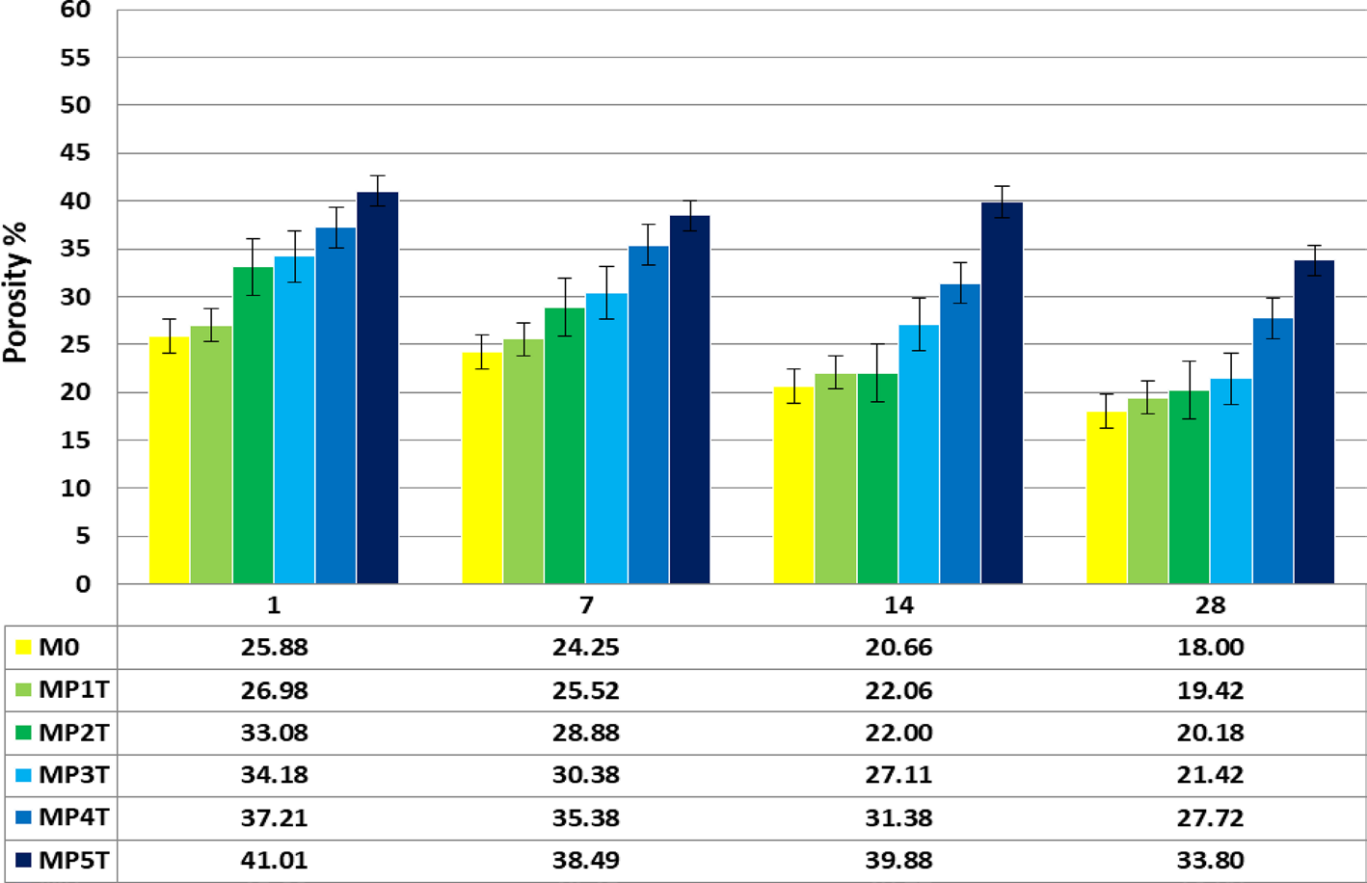
TP of MPxT blends.

In Fig. 11, it is depicted how 1.0% NT altered the overall TP of MPxT-composite pasts. TP decreases when the hydration rate increases, e.g. C_3_S, C_2_S, C_3_A and C_4_AF. TP of blends report that the increase of calcium (aluminate & silicate) hydration products generated by the active NT and CH pozzolanic activity are what cause the composite pastes' lower TP outcomes in the MPxT hybridization. TP rises as the open pores of the specimen shrinks, allowing the NT grains and lime to combine and produce C-A-H, C-S–H, and C-ASH respectively. The porosity values of the WC composite e.g. M0, and the MP1T mix is high dense than the other blends^58^. The porosity increases by NPW% addition, as both MP1T and M0 blends shows lower TP value than the MP3T to MP5T mixes due to the impact of the NPW + NT crystallinity shape. The mixtures with 1.0 wt.% NT exceeds the MP1T sample (M0) due to the reductions in the WC/P percent, which enhances the MCS features, the conjugated 0.5–1.0 wt.% NPW + 1.0 wt.% NS has the higher TP, value among the others. The findings of the MP1T mixes shows a reduction in the results of MCS, and density when the WC is replaced with 1.5 to 2.5% NPW + 1.0% NT.

### Microstructure

**Figure 12:**
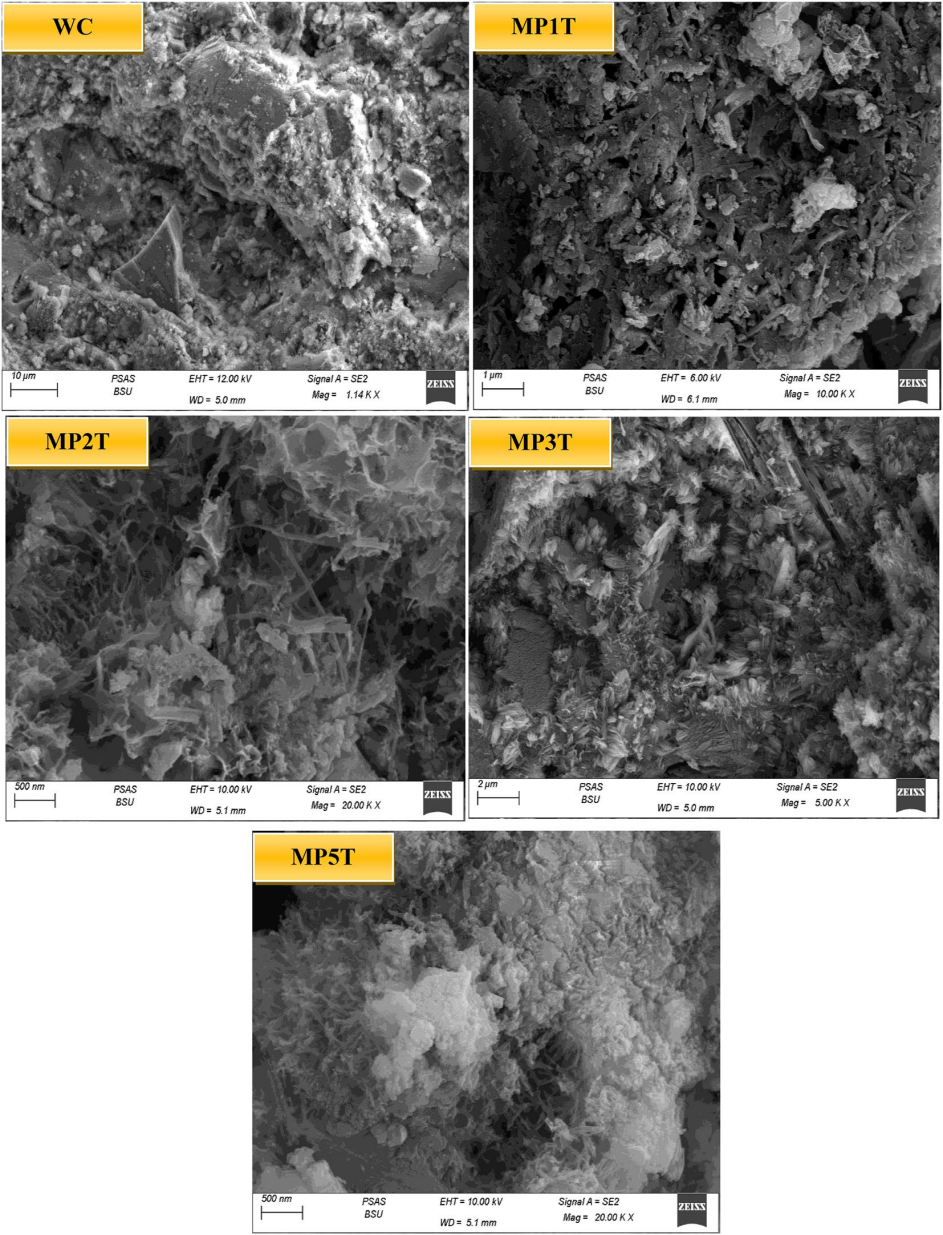
Microstructure of MPxT blends hydrated for 28 days.

The SEM and microstructure of the 28-day-cured MPxT cement composite are shown in Fig. 12. The images show many layers of well-arranged hexagonal pattern from C-H crystals and a high concentration of crystalline hydrates, which together provide a clearly defined porosity structure. The crosslinking of an overabundance of fiber-gel C-S-H hydrates over the course of 28 days’ results in a solid, closed cement structure. As MP1T mix is the optimal percent for the NT as active dynamic filler material, the crystalline C-S-H exhibits needle-like particles and layers of well-organized spherical texture of fiber-gel C-H crystals are increased. In contrast, these crystalline and fibrous layers are decreased with high NT replacement as its act as SCMs has slightly better dynamic features than the inert fillers. The blends that has been morphologically hardened has a squamous microstructure with numerous pores that are filled with nanoparticles. These products have an impact on the specimen's mechanical strength by lowering its TP, which reflects the hydraulic effect of NT. Substitution up to 1.0 wt% NPW has a less dense matrix structure, which could be explained by NT's worse hydraulic capabilities at higher replacement ratios^59^.

### XRD and phase composition

**Figure 13:**
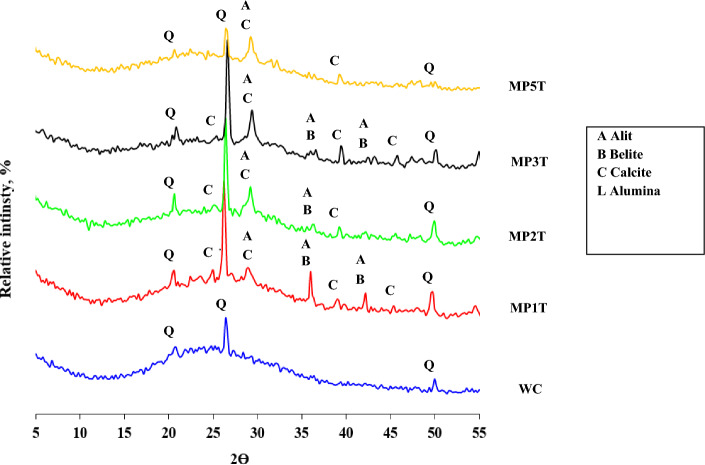
Phase compositions and XRD of MPxT blends hydrated for 28 days.

Figure 13 displays the XRD patterns and hydrated phases of the MPxT and WC blends after a 28-day hydration period. Reduced WC content is responsible for the decrease in residual Portlandite content in MPxT-blended cement composite paste. In MPxT-blend pastes, the amount of the anhydrous larnite (C2S), hatrurite (C3S), W, Ca^2+^, C-S-H, and ettringite falls when the hydraulic action of the NT reacts with portlandite to release it from the cement's hydration kinetics and create more hydrates. All blends had distinct quartz peaks that increased their strength and fire resistance, but increasing density also increased their MCS. The XRD pattern shows layers of organized hexagonal texture from C-S-H crystals and a high concentration of crystalline hydrates, which together create a clearly defined porosity structure. The excess fiber-gel CSH hydrates visible in the XRD pattern are what crosslink a solid, closed cement structure with a longer cure time of 28 days. The crystalline calcium silicate hydrate exhibits layers of fiber-gel with an ordered hexagonal pattern and road-like particles. C–H crystals are increased in MP1T mix as it's the optimal percent value for the NPW incorporated with 1.0 wt.% NT as active hydraulic filler material (see microstructure section). The specimen after it has been hardened displays squamous peaks and numerous pores filled with nanoparticles. These features increase the specimen’s MCS by reducing TP, which reflects the dynamic action of NT.

## Progressing towards sustainability

To protect natural resources and ecosystems from degradation and ensure equality, economic growth, peace and justice for all. The study aimed to identify a new method for disposing of plastic waste and recycling it to use it as a raw material for white cement without compromising its properties and degree of whiteness, while preserving environmental life and sustainable development in the high-density energy industry (white cement production). Environmental waste also causes the death of millions of free-living organisms annually. Plastic waste also causes soil pollution by forming an insulating layer between the soil and plants and their roots. It is also considered dangerous Recycling plastic waste in the cement industry contributes to preserving the environment to a very high degree and helps create job opportunities by attracting companies and opening branches. It also helps clean the environment, in addition to sustainable development. One of the most important ways to dispose of plastic waste is to recycle it in industries to ensure that it does not return to the environment again. Hence, a decrease in total porosity, reflective albedo (Ry) and early rapid expansion. Ultimately, the results may lead to a reduction in the strength of the epidemic, especially in the external environment, and other epidemics.

## Conclusion

The hydration kinetics of WC composites, effective impact of NPW incorporated with 1.0 weight percent NT was assessed. The findings show that using NPW + NT filler to partially replace white cement causes a reduction in Ry, which delays the TP and ST of blends. However, due to the hydraulic dynamic impact of NT, which substitutes clinker functioning as active dynamic nuclei site and increases the yelled of C-S-H hydrate products, water content, and soundness. MPxT pastes have higher MCS, com in contrast, C3S and C2S were found in MPxT-blends lately hydrated, indicating that NT effect promote the hydration rate of C-S-H. Portalndite was detected in NT blends hydrated for 14 days, shows the active nuclei site effect of nano-titania at earlier ages of hydration The initial microstructure of C3S and C2S changes as hydration progresses. In order to achieve the best performance from cement mixes, this current study suggests an optimal content of NT wt.% and PW wt.%, nanoparticles that does not exceed 1.0 wt.% as a partial substitution for both fillers. Ultimately, the proposed mixtures are green mainly due to the reduction of CO_2_ emissions, the reduction of energy used during the production process and the use of raw materials that are treated as “PW” in construction for their production. All of these features are focused on reducing global warming and reducing the amount of plastic waste resulting from the installation of these green white cement materials. However, it is worth considering studying the carbon footprint of white cement production due to conflicting literature values.

## Data Availability

The datasets used and/or analyzed during the current study available from the corresponding author on reasonable request.

## Abbreviations

WC: White cement
PPE: Personal protective equipment
PW: Plastic waste
NPW: Nano plastic waste
NT: Nano Titania
BT: Billion tons
SCMs: Supplemental cementitious materials
C2S: Calcium di-silicate
C3S: Calcium tri-silicate
Pa: Pascal measuring unit
TEM: Transmission electron microscope
SEM: Scanning electron microscope
XRF: X-rays fluorescence
MPxT: Cement paste composition Pw % incorporated with Nano-Titina 1.0%
ASTM: American stander test procedures
Ry: Whiteness reflection
HL: Hinter L (whiteness intensity)
Ha: Hinter a (green color intensity)
Hb: Hinter b (yellow color intensity)
MCS: Mechanical compressive strength
W/CP: Water/cement powder ratio
C-S–H: Tobermorite gel
ST: Setting time
Na_eq_: Sodium equivalent
TP: Total porosity
XRD: X-rays diffractometer
